# The Treatment of Heart Failure in Patients with Chronic Kidney Disease: Doubts and New Developments from the Last ESC Guidelines

**DOI:** 10.3390/jcm11082243

**Published:** 2022-04-17

**Authors:** Matteo Beltrami, Massimo Milli, Lorenzo Lupo Dei, Alberto Palazzuoli

**Affiliations:** 1 Cardiology Unit, San Giovanni di Dio Hospital, Via Torregalli 3, 50142 Florence, Italy; massimo.milli@uslcentro.toscana.it; 2 Cardiology, Department of Life, Health and Enviromental Sciences, University of L’Aquila, 67100 L’Aquila, Italy; lupodei94@gmail.com; 3 Cardiovascular Diseases Unit, Cardio Thoracic and Vascular Department, Le Scotte Hospital, University of Siena, 53100 Siena, Italy; palazzuoli2@unisi.it

**Keywords:** heart failure, chronic kidney disease, estimated glomerular filtration rate, sodium glucose linked transporters 2 inhibitors, treatment, angiotensin receptor blocker neprilysin inhibitors

## Abstract

Patients with heart failure (HF) and associated chronic kidney disease (CKD) are a population less represented in clinical trials; additionally, subjects with more severe estimated glomerular filtration rate reduction are often excluded from large studies. In this setting, most of the data come from post hoc analyses and retrospective studies. Accordingly, in patients with advanced CKD, there are no specific studies evaluating the long-term effects of the traditional drugs commonly administered in HF. Current concerns may affect the practical approach to the traditional treatment, and in this setting, physicians are often reluctant to administer and titrate some agents acting on the renin angiotensin aldosterone system and the sympathetic activity. Therefore, the extensive application in different HF subtypes with wide associated conditions and different renal dysfunction etiologies remains a subject of debate. The role of novel drugs, such as angiotensin receptor blocker neprilysin inhibitors and sodium glucose linked transporters 2 inhibitors seems to offer a new perspective in patients with CKD. Due to its protective vascular and hormonal actions, the use of these agents may be safely extended to patients with renal dysfunction in the long term. In this review, we discussed the largest trials reporting data on subjects with HF and associated CKD, while suggesting a practical stepwise algorithm to avoid renal and cardiac complications.

## 1. Introduction

**Figure 1:**
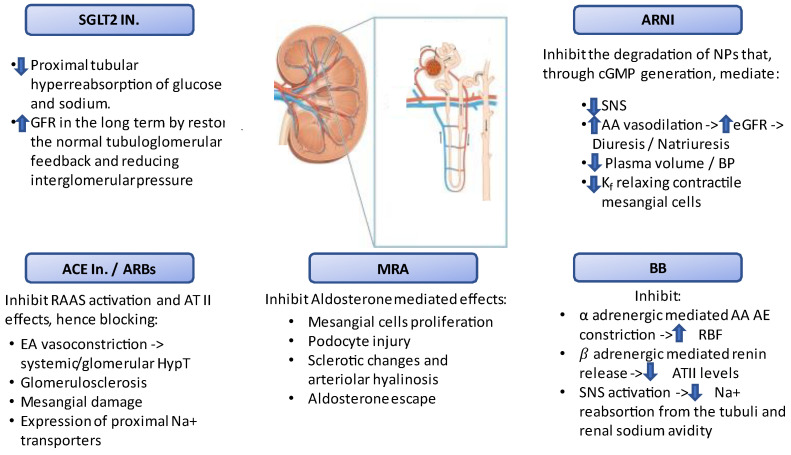
The effects of heart failure drugs on renal physiology. AA: afferent arteriole; ACE In.: angiotensin converting enzyme inhibitors; ARBs: angiotensin receptor blockers; ARNI: angiotensin receptor neprilisin inhibitor; ATII: angiotensin II; BB: beta blockers; BP: blood pressure; cGMP: cyclic guanosine monophosphate; eGFR: estimated glomerular filtration rate; EA: efferent arteriole; HypT: hypertension; K_f_: glomerular capillary ultrafiltration coefficient; MRA: mineralcorticoid receptor antagonist; NPs: natriuretic peptides; RAAS: renin angiotensin aldosterone system; RBF: renal blood flow; SGLT2 In.: sodium glucose transporter protein 2 inhibitors; SNS: sympathetic nervous system.

The most recent HF guidelines propose a revised algorithm for the treatment of heart failure with reduced ejection fraction (HFrEF), with the “quadruple therapy” approach with the use of SGLT-2 inhibitors, angiotensin receptor blocker neprilysin inhibitors (ARNI) (as a replacement of angiotensin-converting enzyme inhibitors (ACE-I) and angiotensin receptor blockers (ARBs) or in de novo HFrEF patients with class of recommendation IIb), on top on B-blockers, and mineralocorticoid receptor antagonists (MRAs), with a substantial improvement in clinical outcomes in terms of hospitalization and mortality [1]. However, renin angiotensin system (RAAS) inhibitors, MRAs, angiotensin receptor blocker neprilysin inhibitors (ARNI), and sodium glucose linked transporters 2 (SGLT2) inhibitors significantly impact the renal function due to changes in renal physiology. These drugs reset the renal function curve, affecting the intraglomerular hydrostatic pressures–natriuresis relationship through the tubule-glomerular feedback mechanism and by contrasting the effects on the afferent and efferent glomerular arteriola induced by different agents. These effects modify the physiological filtration fraction, have different baroceptorial and chemotactic repercussion on the macula densa, and may impact the tubular function (Figure 1). The concomitant use of RAAS inhibitors, MRAs, and novel drug such as SGLT2 inhibitors and ARNI may amplify the process of transitory renal impairment occurring after the early administration, resulting in the inertia of the start and up-titration of these lifesaving therapies. In most of cases, renal impairment is transitory, and the kidney function tends to return to its prior conditions or remain stable in the long term [2]. However, the effect on the renal function induced by polytherapy is not being sufficiently analyzed. Therefore, HF patients with concomitant renal dysfunction are less likely to receive guideline-recommended therapies, even though this is not always justified. In this review, we reported the effects on the kidney of heart failure (HF) drugs in patients with HF and chronic kidney disease (CKD), and we suggested the correct application of these lifesaving therapies in clinical practice.

## 2. Clinical Characteristics of Patients with Chronic Kidney Disease and Heart Failure

Previous studies on outpatients with chronic HF showed that one of the highest prevalence among the non-cardiovascular comorbidities was related to a renal failure ranging from 30% to 50% [3]. The heart and kidneys were strictly related; the dysfunction of either of those organs led to a functional deterioration of the other due to various mechanisms, such as inflammation, oxidative stress, impaired hydro-saline homeostasis, and diuretic resistance [4,5]. In chronic HF, there was decreased cardiac output, predominantly due HFrEF results in decreased organ perfusion. In patients with HFpEF, elevated filling pressures were the main hemodynamic feature and decreased systolic filling resulted in inadequate stroke volume reserved, ultimately causing a decreased cardiac output. A reduction in cardiac output in patients with chronic HF has been shown to result in a decrease in renal blood flow. Additionally, in response to a diminished cardiac output, the kidney promotes mechanisms that result in water and sodium retention, ultimately causing subclinical congestion, which in turn causes further kidney dysfunction. Both in experimental settings and in patients with either chronic or acute HF, an increase in central venous pressures or abdominal pressure was associated with an increased risk of worsening renal function. In cardiorenal syndrome type 2, CKD has been observed in 45 to 63% of patients. Renal congestion, hypoperfusion, and increased right atrial pressure represent hallmarks of this clinical condition [6]. HF and CKD patients shared a poor quality of life and showed a high burden of cardiovascular (CV) risk due to several common risk factors, such as diabetes, hypertension, and coronary artery disease (CAD) [7]. Phenotyping patients with renal dysfunction remains a real challenge; the pathophysiological mechanisms and the prognostic role of renal dysfunction may differ across HFrEF, HFmrEF, and HFpEF. CKD is often associated with more severe HF conditions and stages, independently of left ventricular ejection fraction (LVEF). The relationships between CKD, older age, female sex, diabetes, and HF stage were similar in the three HF groups, but several studies demonstrated that CKD was more prevalent in heart failure with preserved ejection fraction (HFpEF) than in heart failure with mildly reduced ejection fraction (HFmrEF) and HFrEF [8,9]. Other studies showed a higher prevalence of CKD in HFrEF patients [10]. The association between HFpEF and the deterioration of the renal function was independent of the presence of CKD at baseline. Renal dysfunction in HFpEF may be considered a major comorbidity, with a general prognostic impact without any relation with a worse HF status: conversely, in HFrEF patients, kidney dysfunction may reflect the progression of HF, perhaps due to low cardiac output, hemodynamic hypoperfusion, and sympathetic and neurohormonal activation [11].

Among non-CV comorbidities, CKD was the disease more frequently associated with hospitalization [12]. Renal dysfunction, regardless of its definition and screening method, conferred a clinically significant risk for excess mortality in patients with HF [13]. CKD was associated with worse outcomes in all HF phenotype; however, the literature on mortality in HFpEF and CKD shows conflicting results. In the larger meta-analyses, which included a cohort of HFpEF patients, CKD was a more powerful predictor of death [14]. Conversely, a meta-analysis of the Global Group in Chronic Heart Failure (MAGGIC) showed a lower mortality rate and a lower association between CKD and death in patients with HFpEF than in those with HFrEF [15]. This result was confirmed in the Swedish Heart Failure registry, in which the association between CKD and mortality risk was less pronounced in HFpEF patients [16].

In patients with acute heart failure (AHF), we can discern between two distinct phenotypes: patients with baseline renal dysfunction, defined as CKD, and patients developing worsening renal function (WRF) during hospitalization [17]. A new classification of WRF has been proposed, according to the time frame resolution or persistence. The first clinical scenario was a patient with good renal function and occurrence of a “pseudo” WRF during hospitalization for acute HF, that was considered secondary to the decongestion therapy. The increase of in-hospital creatinine did not usually persist after discharge, without consequences for the prognosis if the patient was well treated, with efficient decongestion at discharge. The second scenario was a patient with true WRF due to congestion (increased renal venous pressure) and hypoperfusion (reduced arterial perfusion), in which renal deterioration persisted, with an increase in creatinine also in the post-discharge period and with a higher burden of HF re-hospitalization [18]. Finally, in the third scenario, WRF could occur in the presence of CKD related to reduced cortical blood flow and chronic glomerulosclerosis with reduced cortical wall. This subtype was common in older patients with several comorbidities, where WRF reflected the real deterioration of the renal function, with worse prognostic value. Current classification was uncompleted, because it did not account for serial kidney evaluation after discharge and the severity of an effective estimated glomerular filtration rate (eGFR) impairment (Table 1).

## 3. Therapeutic Target and Limitations in Patients with Heart Failure and Chronic Kidney Disease

All drugs used in HF patients have potentially detrimental effects on the renal function, and they expose HF patients with renal dysfunction to a greater risk of adverse renal complications, such as hyperkalemia and dialysis. Historically, data from randomized controlled trials on the effect of HF medications in HF patients and CKD were limited, due to the exclusion of patients with CKD.

The studies of left ventricular dysfunction (SOLVD) trial enrolled 36% of patients with CKD and eGFR < 60 mL/min/1.73 m^2^; 33% of all patients presented a >0.5 mg/dL increase in serum creatinine; in the final analyses, the benefits on all-cause mortality were maintained across the entire CKD spectrum [19]. This finding was confirmed by the survival and ventricular enlargement (SAVE) trial, which demonstrated the improvement in survival and reduced morbidity in patients with asymptomatic left ventricular dysfunction treated with captopril vs. placebo regardless of CKD (exclusion criteria Cr > 2.5 mg/dL, 33% of patients with CKD). After 42 months of follow-up, the risk for death associated with renal events was hazard ratio (HR) 1.63 (95% CI 1.05–2.52) in the placebo group, versus HR 1.33 (95% CI 0.81–2.21) in the captopril group (*p* = 0.49 for interaction) [20]. Similar findings were found in the trandolapril cardiac evaluation (TRACE) study group, in which 40% of patients with post-myocardial infarct LV dysfunction had CKD. In this group, trandolapril significantly reduced the risk of CV mortality and HF progression [21]. More recently, in the NETWORK and ATLAS trials, patients with Cr > 2.3 mg/dL and Cr > 2.5 mg/dL were excluded, and no specific therapeutic data on advanced CKD could be extrapolated. The valsartan heart failure trial (Val-HeFT) included the higher percentage of patients with HF and CKD (58% of the entire cohort); valsartan significantly reduced the combined endpoint of mortality and morbidity and improved HF symptoms also in HF patients with CKD [22]. Notably, candesartan in heart failure assessment of reduction in mortality and morbidity (CHARM)-added and CHARM-alternative trials, which included a significant proportion of CKD population, confirmed the previous data. However, patients with more severe CKD (creatinine > 3.0 mg/dL) were excluded. In this study, a significant percentage of patients (7.1%) discontinued the therapy due to an increase in creatinine, in the absence of sufficient data regarding the permanent effect on the renal outcome [23].

The Cox proportional hazards regression models in the SOLVD trial showed that, compared to placebo, ACE-I did not reduce the decline in eGFR, that was similar in both groups. However, the study recommended to avoid the withdrawal of ACE-I in patients with low and moderate eGFR decline due to the beneficial effect on the overall CV outcome [24]. Moreover, both ACE-I and ARBs showed to significantly slow the eGFR decline in diabetes and nephropathy due to their favorable physiological effect [25] (Table 2). 

In patients in sinus rhythm with HF and LVEF < 50%, B-blockers reduced mortality versus placebo without any deterioration in renal function over time in patients with moderate or moderate to severe renal impairment [26]. These beneficial results were lost in patients with HF and atrial fibrillation (AF) at any level of eGFR. Metoprolol was analyzed in three renal function subgroups and demonstrated an effective reduction in all-cause death and hospitalizations for worsening HF in patients with eGFR < 45 mL/min/1.73 m^2^ and eGFR 45 to 60 mL/min/1.73 m^2^, as in those with eGFR > 60 mL/min/1.73 m^2^ [27]. Meta-analyses from the CAPRICORN (carvedilol postinfarct survival control in left ventricular dysfunction study) and COPERNICUS (carvedilol prospective randomized, cumulative survival study) studies showed that carvedilol was well tolerated in patients with and without CKD, with an increased relative incidence in the transient increase in serum creatinine, without serious adverse kidney effects and electrolyte changes in CKD patients. Carvedilol therapy reduced the composite outcome of CV mortality or HF hospitalization, without significant effects on sudden death in the presence of mild to moderate CKD [28]. Carvedilol reduced morbidity and mortality in dialyzed patients with dilated cardiomyopathy [29]. Current contrasting findings show that the use of B-blockers in dialysis or in patients with severe kidney deterioration needs to be further investigated (Table 3).

Historically, MRAs were considered contra-indicated in patients with renal dysfunction, due to the higher risk of hyperkalemia. The beneficial effect of both spironolactone and eplerenone on the outcomes of HF patients has recently extended to those with renal dysfunction; however, no trials focused on the effects of MRAs on the renal outcome and related mortality in patients with HF and eGFR < 30 mL/min/1.73 m^2^ [30]. A recently published secondary analysis of the eplerenone in mild patients hospitalized and survival study in heart failure (EMPHASIS-HF) examined the beneficial and adverse effects of eplerenone on renal function. Even though patients with an eGFR < 50 mL/min/1.73 m^2^ were assigned lower target doses of eplerenone (25 mg versus 50 mg), the drug showed a beneficial effect on the outcome versus placebo; however, patients with eGFR 30–49 mL/min/1.73 m^2^ experienced higher incidences of hyperkalemia, renal failure, and drug discontinuation [31]. Patients with moderate renal dysfunction should be monitored closely after the initiation of a MRAs, with frequent K^+^ analyses and a slower up-titration of therapy, due to the higher risk of hyperkalemia and the potential arrhythmic and renal consequences. MRAs treatment did not affect renal function in subjects without evidence of HF; finerenone, a non-steroidal selective MRA, resulted in a lower risk of CKD progression and CV events than placebo in patients with CKD and type two diabetes [32]. The aforementioned data reinforced the use of MRAs in patients with either HF and mild to moderate CKD, or in patients with high CV risk associated with renal dysfunction, but a larger use in more advanced HF and CKD stages was not extensively carried out, and it deserves specific analyses.

In patients with HF, the beneficial effect of ARNI showed several physiological mechanisms, including the increase in intracellular cyclic GMP that counteracts the constrictive effects of the tubule-glomerular feedback on the afferent arteriole. In a retrospective analysis of the prospective comparison of ARNI with ACE-i to determine impact on global mortality and morbidity in heart failure (PARADIGM-HF) trial, sacubitril and valsartan improved CV outcomes and led to a slower rate of eGFR decline versus enalapril (difference of 0.4 mL/min/1.73 m^2^ per year). The relative risk reduction associated with sacubitril and valsartan was similar in patients with and without renal dysfunction, despite causing a modest increase in the urinary albumin to creatinine ratio [33]. The extent of the benefit was larger in patients with diabetes than those without [34]. This effect was also confirmed in HFpEF patients, in whom sacubitril and valsartan reduced the risk of a ≥50% reduction in eGFR, end-stage renal disease, or death from renal cause, and slowed the decline in eGFR during the follow-up versus valsartan. The renal benefits were more evident in patients with LVEF between 30–60%; however, the entire population enrolled in the study experienced an eGFR reduction of 1.8 mL/min/1.73 m^2^ per year in the sacubitril and valsartan group, versus 2.4 mL/min/1.73 m^2^ per year in the RAAS inhibitors group, regardless of the LVEF [35].

SGLT-2 co-transporters are mainly located in the renal proximal convoluted tubule; by inhibiting Na+ and glucose reabsorption, SGLT-2 inhibitors promote glucosuria and natriuresis and reduce extracellular fluid and plasma volume. These effects reduced the left ventricular afterload and preload and decreased blood pressure and arterial stiffness, while improving the subject-endocardial blood flow [36]. The renal hemodynamic effects of SGLT-2 inhibition were ascribable to the reduction in intra-glomerular pressure. The effect of SGLT-2 to counterbalance the glomerular hypertension and hyperfiltration was crucial in type two diabetes mellitus (T2DM), where hyperglycemia leads to renal Na+ reabsorption, causing an afferent renal vasodilatory response through the tubuloglomerular feedback [37]. Empaglifozin improves the diabetic kidney disease by alleviating mitochondrial fission via AMPK/SP1/PGAM5 pathway [38,39]. With all these favorable effects, SGLT-2 inhibitors led to nephron protection and reduced the progression of diabetic nephropathy. Moreover, sodium-hydrogen exchanger 3 (NHE3) is expressed in proximal tubule and exchanges Na+ into the cell with proton export [40]. NHE3 increases the expression of SGLT-2 in the nephron membrane, leading to sympathetic/RAAS activation and acidosis. The restoration of Na+ homeostasis depends also on the inhibition of renal NHE3 by SGLT-2 inhibitors. Finally, in a meta-analysis of randomized controlled trials, SGLT-2 decreased albuminuria, slowing the progression of microalbuminuria to macroalbuminuria and reducing the risk of end-stage renal disease [41].

In recent years, landmark trials established the CV benefits and renal outcome of SGLT-2 inhibitors in the HFrEF population. The empagliflozin outcome trial in patients with chronic heart failure and a reduced ejection fraction (EMPEROR-Reduced) showed that empagliflozin reduced both CV death and HF hospitalization in patients with HFrEF, despite OMT. The trial included patients with eGFR higher than 20 mL/min/1.73 m², and 48% of the subjects enrolled had an eGFR < 60 mL/min/1.73 m^2^ [42]. Empagliflozin reduced the primary outcome and total number of HF hospitalizations in patients with and without CKD, and had the beneficial effect of reducing the decline of the renal function, regardless of the severity of renal function at baseline [43]. The analyses of the CREDENCE (canagliflozin and renal events in diabetes with established nephropathy clinical evaluation) trial showed the effects of canagliflozin in reducing the incidence of kidney-related adverse events in patients with T2DM and CKD [44]. Moreover, the dapagliflozin and prevention of adverse outcomes in heart failure (DAPA-HF) trial included 41% of patients with eGFR < 60 mL/min/1.73 m^2^ and excluded those with eGFR < 25 mL/min/1.73 m^2^ [45]. The results of the trial showed that the benefits of dapagliflozin on morbidity and mortality in HFrEF did not differ by eGFR category or by examining eGFR as a continuous variable, with a significantly slower rate of decline in eGFR, regardless of the presence of diabetes [46]. In the DAPA-CKD (dapagliflozin and prevention of adverse outcomes in chronic kidney disease) trial, properly designed for patients with CKD, dapagliflozin significantly reduced the decline in eGFR, the end-stage kidney disease, or death from renal or CV causes [47] (Table 4).

In clinical practice, as demonstrated in several trials, the initiation of SGLT-2 inhibitors was associated with an initially mild drop of eGFR over the first weeks. This decrease in eGFR was reversible, and the renal function gradually returned to its baseline levels, with a stabilization of the renal function during the follow-up. The initial mild drop in eGFR should not lead to a premature discontinuation of the SGLT-2 inhibitors treatment.

Recently, novel therapies in HFrEF have been proposed. The vericiguat global study in subjects with heart failure with reduced ejection fraction (VICTORIA) trial demonstrated the effect of vericiguat, a soluble guanylate cyclase stimulator, in reducing the primary composite outcome of CV death or HF hospitalization. For the first time in HF therapies, the study included patients with eGFR higher than 15 mL/min/1.73 m^2^; the beneficial effects of vericiguat were consistent across the entire range of eGFR, irrespectively of WRF [48].

The use of hydralazine and isosorbide dinitrate (H-ISDN) in HFrEF is rarely used in clinical practice. However, treatment with H-ISDN was recommended in the last guidelines for HFrEF patients who are intolerant to RAAS inhibitors, and in African-American HFrEF patients who are symptomatic despite optimal neurohumoral therapy. The treatment with H-ISDN is safe in patients with CKD. However, in a recent trial, H-ISDN on the top of standard medical therapy did not improve exercise capacity in patients with cardiorenal syndrome and HFrEF [49]. These findings are in agreement with real-world data on a large cohort of HFpEF and HFmrEF patients enrolled in the Swedish Heart Failure Registry where patients in the sub-group analyses with HF and CKD (eGFR 30–59 mL/min/1.73 m^2^ and eGFR < 30 mL/min/1.73 m^2^) benefitted from nitrate administration [50].

## 4. Renal Diagnostic Exams and Comparison between Different Criteria

Several methods and diagnostic approaches have been proposed for the evaluation of renal function in chronic conditions. So far, no universal definition and classification exists, and this contributes to complicate the definition and severity of CKD.

The simplified modification of diet in renal disease (MDRD) formula showed a few limitations, such as the use of body mass and age of patients showing an incorrect relationship between serum creatinine and muscle mass variability. The Cockcroft-Gault formula showed the worst accuracy in measuring eGFR; however, it was accurate in improving the risk stratification for death in HF patients, perhaps due to the inclusion of weight in its formula (not included in MDRD) [51]. The simplified modification of diet in renal disease the chronic kidney epidemiology collaboration (CKD-EPI) formula, based on serum creatinine and serum cystatin C, estimated more accurately the real eGFR in all HF patients, particularly in those with preserved or moderately impaired renal function [52,53]. Cystatin C concentration was less affected by age, sex, muscle mass, or diet than creatinine. In detail, CKD-EPI_crea/cys_ and CKD-EPI_cys_ (CKD-EPI creatinine and cystatin formula: 177.6 × (serum creatinine (mg dL)) − 0.65 × (serum cystatin C (mg L)) − 0.57 × age − 0.2) provided less bias and more accurate estimates of eGFR than CKD-EPI_crea_ [54]. Recently, the new European Kidney Function Consortium equation showed improved accuracy and precision with lower age-related bias compared with the commonly used equations for estimating GFR from serum creatinine (SCr) levels [55].

The limitation of eGFR and creatinine in assessing renal function should lead to the addition of several marker and laboratory exams, in order to deeply monitor renal function. Blood urea nitrogen (BUN) was commonly assessed in association with renal function and reflected glomerular filtration, tubular reabsorption, and neurohormonal activation. The main difference between sCr and BUN was the reabsorption of BUN at the tubular level. Recently, the BUN to creatinine ratio was able to differentiate pre-renal and intrinsic renal diseases; in particular, neurohormonal activation led to a disproportional reabsorption of BUN in comparison with creatinine. Both BUN and the BUN to creatinine ratio identified HF patients with an increased risk of adverse outcomes. Moreover, the urine BUN to creatinine ratio predicted diuretic efficiency and a significant difference for HF rehospitalization and death rate at 180 days [56]. Albuminuria was mainly a marker of increased glomerular permeability and failure of tubular reabsorption, and affected around 20–30% of patients with HF, particularly those with associated CKD. Albuminuria was a marker of endothelial dysfunction, inflammation, podocyte damage, disrupted tubular reabsorption, and congestion, and provided additional information regarding the mechanism of renal impairment on top of the eGFR or BUN to creatinine ratio [57]. Micro- and macro-albuminuria were associated with increased mortality in the HF population, independently from eGFR, thus highlighting the concept that albuminuria itself could accelerate the progression of renal dysfunction via an impairment in the recovery cells in Bowman’s space and a chronic overload and damage to the megalin cubilin transporter system in the proximal tubule.

The tubulo-interstitial injury in HF, as measured by increased urinary neutrophil gelatinase-associated lipocalin (NGAL) concentrations, may indicate renal damage, even in the presence of normal glomerular filtration. Poniatowski et al. had recognized serum and urine NGAL as sensitive early markers of renal dysfunction in patients with chronic HF and normal serum creatinine but reduced eGFR [58]. In detail, the extension of tubular damage was related to increased urinary concentrations of three urinary markers of tubular damage: NGAL, *N*-acetyl-beta-D-glucosaminidase (NAG), and kidney injury molecule 1 (KIM-1). The increases in these tubular markers were related to a poorer outcome in HF patients, even when eGFR was normal.

Recently, urinary sodium—assessed in spot urinary samples—showed interesting data in both acute and chronic HF patients; measuring natriuresis early after hospitalization could reliably identify patients with a poor diuretic response during hospitalization, who might require an adjustment of their diuretic strategies [59]. In a single-center study of HF outpatients, a drop in urinary spot sodium concentration was found a week before hospitalization for HF. The outpatient assessment of spot urinary sodium may therefore be a readily applicable marker to guide or initiate treatment and prevent hospitalization for AHF [60]. The etiology of hypochloremia in patients with HF was not only related to the diuretic used, but was also associated with the activation of RAAS and a stimulatory effect on the with-no-lysine kinases, which may increase the renal sodium-chloride co-transporter activity [61]. A sub-analysis of the beta-blocker evaluation of survival (BEST) trial showed that both urinary hypochloremia and hyponatremia were related to a poor prognosis in HF patients, suggesting the routine use of spot urinary samples to monitor the renal response and adjust the treatment of HF [62].

## 5. Potential Strategy for the Correct Use of Neuro-Hormonal Inhibition Treatments According to Renal Dysfunction Severity

Historically, CKD represents a real “nightmare” when tailoring and optimizing the HF therapy. Although the latest ESC guidelines recommended the concomitant use of four agents after the diagnosis of HF, the potential treatment strategy across CKD spectrum was not elucidated. Based on the analysis of a larger trial evaluating the sympathetic antagonism and the treatment with RAAS inhibitors, the use of common neuro-hormonal inhibitory therapy was recommended in mild to moderate CKD, even if some studies seemed to suggest a protective role of B-blockers in patients with more severe renal dysfunction. The new ESC guidelines recommended the quadruple therapy in patients with eGFR > 60 mL/min/1.73 m^2^. SGLT-2 inhibitors were recommended for all patients with HFrEF in addition to ACE-I/ARNI, a beta-blocker, and an MRA. This combined approach may suddenly change the renal physiology, which could lead to a higher risk of a progressive decline in eGFR, even in patients with normal renal function. In those patients, a careful monitoring of the renal function and electrolytes should be performed 3 or 4 weeks after the start of the therapy, in order to avoid sudden eGFR deterioration and potassium (K^+^) increase. In patients with eGFR 30–60 mL/min/1.73 m^2^, a triple therapy with low dose B-blocker, RAAS inhibitors, or ARNI and a full dosage of SGLT-2 inhibitors should be prescribed. During follow-up, we can add low-dose MRAs if creatinine levels remain stable—or increase by less than 30%—and if K is < 5 meq/L. More attention should be paid in patients with eGFR 15–30 mL/min/1.73 m^2^, where we suggest starting with low-dose B-blockers and SGLT-2 inhibitors, adding RAAS inhibitors after the up-titration of the first two agents only if creatinine increases by <30% or K^+^ is < 5 mmol/L. In patients with severe renal dysfunction, the multi-drug approach may become deleterious, and the administration of lower dosages of B-blocker with the subsequent addition of ACE-I without up-titration may be considered.

**Figure 2:**
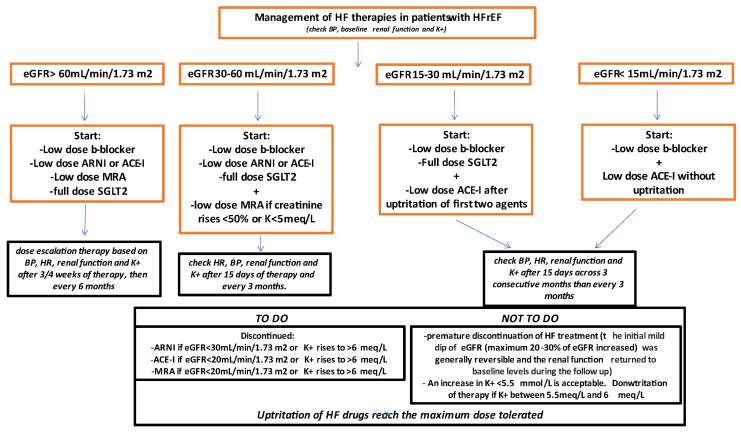
Management of HF therapies in patients with HFrEF. HFrEF: Heart Failure with reduced ejection fraction; ACE-I: angiotensin converting enzyme inhibitor; ARNI: angiotensin receptor neprilisin inhibitor; B-Blocker: beta blocker; BP: blood pressure; eGFR: estimated glomerular filtration rate; HR: heart rate; K^+^: potassium; HF: heart failure MRA: mineralcorticoid receptor antagonist; SGLT2: sodium glucose late transporter 2 inhibitors.

Overall, in patients with renal dysfunction, we recommend checking renal function and K^+^ after 15 days of starting therapy and then every 2 to 3 months in order to reach the maximum tolerated dose. If serum creatinine increases by >50% or above 3.5 mg/dL, treatment should be discontinued. Hyperkalemia was the most frequent cause for drug discontinuation; the down-titration of HF drugs was recommended if K^+^ was between 5.5 meq/L and 6 meq/L, and temporary discontinuation was advised if potassium was above 6 meq/L (Figure 2). When adjusting for the discontinuation of ACE-I/ARB, hyperkalemia was no longer associated with mortality, suggesting that it may be a risk marker for the discontinuation of ACE-I/ARB rather than a risk factor for worse outcomes. In patients with normal renal function and isolated K^+^ increase, novel K^+^ binder such as patiromer and sodium zirconium cyclosilicate substantially reduced serum K^+^ levels in the long term, allowing up-titration and the maintenance of the RAAS inhibitors and ARNI therapy. Therefore, both agents have been safety tested in patients with chronic HF as providing beneficial effects on the CV risk [63].

Therefore, combining current HF lifesaving drugs significantly improved the hard endpoints in the HF population; thus, the aim was to use a sequential up-titration of single agents while checking renal function, electrolytes, and blood pressure, thus avoiding the risks of treatment side effects.

## 6. Conclusions

CKD in HF is associated with a worse prognosis across the entire eGFR spectrum. The recently proposed HF “quadruple therapy” significantly reduces mortality and HF hospitalization also in patients with HF and CKD. Despite the favorable effects of these HF medications, specific studies investigating the effect of the treatment with an eGFR lower than 30 mL/min/m^2^ remain scarce, and their safety should be confirmed over a long observational period. Conversely, the false myth of administering inadequate target dose or withdrawing HF therapies to avoid end-stage renal disease resulted in a lower use of these lifesaving therapies, with a significant impact on the HF prognosis. The extensive application of multiple HF agents needs caution and a frequent monitoring of specific laboratory patterns, with particular attention during the titration phase and the recurrence of HF.

## Figures and Tables

**Table 1:** Clinical scenarios and RIFLE (risk of renal failure, injury to the kidney, failure of kidney function, loss of kidney function, end-stage renal failure) criteria and AKIN (acute kidney injury network) criteria for diagnosis of acute kidney injury.

Clinical Scenarios		
(1) “Pseudo” WRF	Good renal function at baseline and occurrence of WRF during hospitalization for acute HF, usually secondary to the decongestion therapy.	
(2) “True” WRF	WRF due to congestion and hypoperfusion, in which renal deterioration persisted also in the post-discharge period with a higher burden of HF re-hospitalization.	
(3) WRF in CKD	WRF could occur in the presence of CKD. This subtype was common in older patients with several comorbidities, where WRF reflected the real deterioration of the renal function, with worse prognostic value.	
**Laboratory/urine Output Criteria**		
	eGFR Criteria	Urine output criteria
RIFLE (an acute rise in SCr over 7d)		
Risk	Increased SCr ≥ ×1.5 or eGFR decrease > 25%	UO < 0.5 mL/kg/h × 6 h
Injury	Increase in SCr ≥ ×2 or eGFR decrease > 50%	UO < 0.5 mL/kg/h × 12 h
Failure	Increase in SCr ≥ ×3 or eGFR decrease > 75% or SCr ≥ 4.0 mg/dL	UO < 0.5 mL/kg/h × 24 h or anuria × 12 h
Loss	Persistent ARF = Complete loss of kidney function > 4 wk	
ESKD	End stage renal disease (>3 months)	
AKIN (an acute rise in SCr within 48 h)		
Stage 1	Same as RIFLE Risk or increase in SCr ≥ 0.3 mg/dL (≥26.4 μmol/L)	Same as RIFLE Risk
Stage 2	Same as RIFLE Injury	Same as RIFLE Injury
Stage 3	Increase in SCr ≥ ×3 or serum creatinine of ≥4.0 mg/dL with an acute increase of at least 0.5 mg/dL or RRT	Same as RIFLE Failure

WRF: worsening renal function; CKD: chronic kidney disease; HF: heart failure; AKIN: acute kidney injury network; ARF: acute renal failure; d: days; ESKD: end-stage kidney disease; eGFR: estimated glomerular filtration rate; h: hour; RIFLE: risk of renal failure, injury to the kidney, failure of kidney function, loss of kidney function, and end-stage renal failure; RRT: renal replacement therapy; SCr: serum creatinine; UO: urine output; and wk: weeks.

**Table 2:** Comparison in renal function outcome between trials evaluating therapy with ACE-I, ARBs, and MRAs in HF patients.

Trial; Author; Year	Pts (*n*)	Design	Main Eligibility Criteria	Primary Outcome	Mean Follow up (years)	Renal Function Exclusion	CKD Groups (eGFR, mL/min/ 1.73 m^2^)	Main Findings
**Angiotensin Converting Enzyme inhibitors**								
CONSENSUS; 1987; The CONSENSUS Trial Study Group	253	Enalapril vs. Pl.	Congested HF, NYHA IV, cardiomegaly on chest X-ray	ACM	0.5	Serum creatinine concentration > 3.4 mg/dL	NA	Enalapril significantly reduced ACM in patients with sCr > 1.39 mg/dL compared to pl. (30% vs. 55%) but did not have a significant effect in those with sCr < 1.39 mg/dL.
SOLVD treatment; 1991; The SOLVD Investigators [19]	2569	Enalapril vs. Pl.	LVEF ≤ 35%, NYHA I–IV (90% NYHA II, III)	ACM	3.4	Creatinine > 2 mg/dL	≥60 (*n* = 1466) (59, 7%) <60 (*n* = 1036) (40, 3%)	Enalapril reduced mortality and hospitalization in SHF patients without significant heterogeneity between those with and without CKD.
SOLVD prevention; 1992; The SOLVD Investigators	4228	Enalapril vs. Pl.	Receiving digitalis, diuretics, or vasodilators (remainder same as SOLVD treatment trial)	ACM	3.08	Creatinine > 2 mg/dL	<45 (*n* = 450) 10.6% ≥45 <60 (*n* = 669) 15.8% ≥60 <75 (*n* = 640) 15.1% >75 (*n* = 863) 20.4	No significant interaction between CKD and treatment
SAVE; 1992; Tokmakova et al. [20]	2331	Captopril vs. Pl.	Acute myocardial infarction (age 21–80 years) LVEF < 40%	ACM	3.5	Creatinine > 2.5 mg/dL	≥60 (*n* = 1562) 67% <60 (*n* = 769) 33%	Captopril reduced CV events irrespective of baseline kidney function. CKD was associated with a heightened risk for all major CV events after MI, particularly among subjects with an eGFR < 45 mL/min/1.73 m^2^.
AIRE; 1997; Hall et al.	2006	Ramipril vs. Pl.	Acute myocardial infarction (ECG and enzymes) and transient or persistent congestive heart failure after index infarct. Clinical CHF by physical examination or radiography.	ACM	1.25	NA	NA	ACM significantly lower for Ramipril (17%) than pl. (23%).
TRACE; 1995; Køber et al. [21]	1749	Trandolapril vs. Pl.	Able to tolerate a test dose of 0.5 mg trandolapril adults with acute myocardial infarction 2–6 days prior to trial entry. Echocardiographic ejection fraction < 35%	ACM	3	Creatinine > 2.5 mg/dL	NA	Trandalopril reduced relative risk of death. Trandolapril also reduces the risk of death from CV causes.
NETWORK; 1998; The NETWORK investigators	1532	Enalapril 2.5 vs. 5 vs. 10 mg BID	Age 18 to 85 years, NYHA II–IV, abnormality of the heart and current treatment for heart failure	ACM, HFH, WHF	0.5	Creatinine > 2.3 mg/dL		No relationship between dose of enalapril and clinical outcome in patients with HF.
ATLAS; 1999; Packer et al.	3174	Lisinopril high vs. low dose	LVEF ≤ 30 NYHA II–IV	ACM	3.8	Creatinine > 2.5 mg/dL	Creatinine > 1.5 mg/dL 2176 (68.5%) Creatinine < 1.5 mg/dL 998 (31.5%)	ACM was non-significantly reduced both in patients with and without CKD.
**Angiotensin Receptor Blockers**								
Val-HeFT; 2003; Carson et al. [22]	5010	Valsartan vs. Pl.	LVEF < 40%; clinically stable CHF NYHA II–IV; treatment with ACE inhibitors; LVDD > 2.9 cm/bsa	ACM	1.9	Creatinine > 2.5 mg/dL	<60 2114 (47%) ≥60 2196 (53%)	Patients with WRF demonstrated the same benefits with valsartan treatment compared with pl. in the overall population.
CHARM added, 2001; McMurray et al. [23]	2548	Candesartan vs. Pl.	LVEF ≤ 40%; NYHA II–IV; treatment with ACE inhibitor	CV death or HFH	3.4	Creatinine >3 mg/dL	≥60 67% <60 33%	The risk for CV death or hospitalization for worsening CHF as well as the risk for ACM increased significantly below an eGFR of 60 mL/min per 1.73 m^2^.
CHARM alternative, 2003; Granger et al.	2028	Candesartan vs. Pl.	CHF NYHA II–IV, LVEF ≤ 40%, ACE inhibitors intolerance	CV death or HFH	2.8	Creatinine > 3 mg/dL	≥60 57.4% <60 42.6%	See above
HEEAL; 2009; Konstam et al.	3846	High dose vs. Low dose Losartan	LVEF ≤ 40%; NYHA II–IV; ACE inhibitors intolerance	ACM or HFH	4.7	Creatinine > 2.5 mg/dL	NA	Losartan 150 mg vs. 50 mg maintained its net clinical benefit and was associated with reduced risk of death or HFH, despite higher rates of WRF and greater rates of eGFR decline.
**Mineralcorticoid Receptor Antagonist**								
RALES; 1999; Kulbertus et al.	1663	Spironolactone vs. Pl.	LVEF < 35%; NYHA III–IV; creatinine ≤ 2.5 mmol/L	ACM	2	creatinine ≥ 2.5 mg/dL	<60 (*n* = 792) 47.62% ≥60 (*n* = 866) 52.07%	Individuals with reduced baseline eGFR exhibited similar relative risk reductions in all-cause death and the combined. Endpoint of death or hospital stayed for HF as those with normal renal function and greater absolute risk reduction compared with those with a higher baseline eGFR.
EMPHASIS-HF, 2001; Zannad et al. [30]	2737	Eplerenone vs. Pl.	LVEF ≤ 35%; NYHA II; eGFR ≥ 30 mL/min/1.73 m	CV death or HFH	1.75	eGFR < 30 mL/min/1.73 m	<60 (*n* = 912) 33.32% ≥60 (*n* = 1821) 66.53%	Eplerenone, as compared with placebo, reduced both the risk of death and the risk of hospitalization in HFrEF patients with CKD.
TOPCAT; 2021; Khumbanj, et al.	3445	Spironolactone vs. placebo (*n* = 3445)	LVEF ≥ 45%; HF hospitalization or elevated NP level; eGFR ≥ 30 mL/min/1.73 m^2^ or creatinine ≤ 2.5	CV death or aborted cardiac arrest or hospitalization for HF	3.3	eGFR < 30 mL/min/1.73 m or serum creatinine >2.5 mg/dL	<45 (*n* = 411) 11.9% 45–60 (*n* = 533) 15.47% ≥60 (*n* = 823) 23.88%	The primary endpoint was similar between the spironolactone and placebo arms. The risk of adverse events was amplified in the lower eGFR categories. These data supported use of spironolactone to treat HFpEF patients with advanced CKD only when close laboratory surveillance was possible.

ACE: angiotensin-converting enzyme inhibitor; ACM: all-cause mortality; CHF: congestive heart failure; CKD: chronic kidney disease; CV: cardiovascular; ECG: electrocardiogram; eGFR: estimated glomerular filtration rate; HF: heart failure; HFH: hospitalization for heart failure; HR: hazard ratio; LVEF: left ventricular ejection fraction; LVDD: left ventricular diastolic diameter; MI: myocardial infarction; NYHA: New York Heart Association; Pts: patients; NA: not available; Pl.: placebo; sCr: serum creatinine; SHF: sever heart failure; WHF: worsening heart failure; and WRF: worsening renal function.

**Table 3:** Comparison in renal function outcome between trials evaluating therapy with Beta Blockers in HF patients.

Trial; Author; Year	Pts (*n*)	Design	Main Eligibility Criteria	Primary outcome	Mean Follow up (years)	Renal Function Exclusion	CKD Groups (eGFR, mL/min/ 1.73 m^2^)	Main Findings
**Beta-Blockers**								
MDC; 1993; Waagstein et al.	383	Metoprolol vs. Pl.	LVEF ≤ 40%; NYHA II, III	ACM	0.9	NA	NA	Treatment with Metoprolol improved symptoms, LVEF, exercise time. It reduced PCWP and clinical deterioration.
CIBIS; 1994; CIBIS Investigators and Committees	641	Bisoprolol vs. Pl.	LVEF ≤ 40% NYHA III–IV Age 18–75 Treatment with diuretic and vasodilator	ACM	1.9	Creatinine > 3.4 mg/dL	Renal insufficiency being a non-inclusion criterion	No significant difference in mortality or sudden death. Improvements in functional status in the bisoprolol arm.
US-Carvedilol; 1996; Packer et al.	1094	Carvedilol vs. Pl.	LVEF ≤ 35%; NYHA II, III despite at least two months of treatment with diuretics and an ACE inhibitor	ACM	0.5	Clinical important renal disease	NA	Carvedilol reduced overall mortality rate, CV risk, hospitalization for CV reasons.
MERIT HF; 1999; MERIT-HF Study Group [27]	3991	Metoprolol vs. Pl.	LVEF ≤ 40%; NYHA II–IV	ACM	1.0	NA	<45 (*n* = 493) ≥ 45 ≤ 60 (*n* = 976) >60 (*n* = 2496)	Metoprolol CR/XL was effective in reducing death and hospitalizations for worsening HF in patients with eGFR < 45 as in those with eGFR > 60. eGFR was a powerful predictor of death and hospitalizations from HF.
CIBIS-II; 1999; CIBIS-II Investigators and Committees	2289	Bisoprolol vs. Pl.	LVEF ≤ 35%; NYHA III, IV	ACM	1.3	Creatinine > 3.4 mg/dL	<60 mL/min (*n* = 849) 37.1% ≥60 mL/min (*n* = 1198) 52.3%	Patients with eGFR <60 mL/min had a markedly higher mortality rate than patients with less compromised renal function; however, they benefited to the same extent from bisoprolol treatment.
COPERNICUS; 2001; Eric J Eichhorn et al.	2289	Carvedilol vs. Pl.	LVEF ≤ 25%; NYHA IV	ACM	0.9	Creatinine > 2.8 mg/dL	≤60 (*n* = 2566) 61% >60 (*n* = 1651) 39% Data to be referred to both the COPERNICUS and CAPRICORN studies considered together	Among the CKD group, treatment with carvedilol was associated with decreased risks of ACM, CV mortality, HF mortality, first HFH. Treatment with carvedilol did not have a statistically significant impact on sudden cardiac death in HF patients with CKD.
CAPRICORN; 2005; McMurray et al.	1959	Carvedilol vs. Pl.	CHF LVEF ≤ 35%	ACM	1.3	Renal Impairment	≤60 (*n* = 2566) 61% >60 (*n* = 1651) 39% Data to be referred to both the COPERNICUS and CAPRICORN studies Considered together.	See Copernicus results above.
COMET; 2003; Pool Wilson et al.	3029	Carvedilol vs. Metoprolol	LVEF ≤ 35%; NYHA II to IV; ACE inhibitor therapy for at least 4 weeks; Diuretic therapy for at least 2 weeks. Prior hospitalization for CV reasons at least once in the year preceding inclusion.	ACM	4.8	NA	NA	Carvedilol improved vascular outcomes better than metoprolol.
SENIORS; 2005; Flather et al.	2128	Nebivolol vs. Placebo	Age ≥ 70 y, HF confirmed as HF hospitalization in recent 12 months and/or LVEF ≤ 35% in recent 6 months	ACM or CV hospitalization	1.8	Significant renal disease	<55.5 (*n* = 704) 33% 55.5–72.8 (*n* = 704) 33% >72.8 (*n* = 704) 33%	SENIORS was not powered to detect reductions in the primary outcome for the renal sub-groups and hence none of the eGFR tertiles reached statistical significance. Nebivolol was safe for use in those with renal dysfunction, albeit with a marginal increase in bradycardia-related treatment discontinuation.

ACM: all-cause mortality; CHF: congestive heart failure; CKD: chronic kidney disease; CV: cardiovascular; eGFR: estimated glomerular filtration rate; HF: heart failure; Pts: patients; HFH: hospitalization for heart failure; LVEF: left ventricular ejection fraction; NYHA: New York Heart Association; pts: patients; NA: not available; PCWP: pulmonary capillary wedge pressure; Pl.: placebo; WHF: worsening heart failure; and WRF: worsening renal function.

**Table 4:** Comparison in renal function outcome between trials evaluating HF therapy with SGLT2 inhibitors, ARNI, and agents considered in selected HFrEF patients.

Trial; Author; Year	Pts (*n*)	Design	Main Eligibility Criteria	Primary Outcome	Mean Follow up (years)	Renal Function Exclusion	CKD Groups (eGFR, mL/min/ 1.73 m^2^)	Main Findings
**Sodium Glucose Linked Transporter 2 Inhibitors**								
DAPA-HF; 2019; Mc Murray et al. [45]	4744	Dapaglifozin vs. Pl.	LVEF ≤ 40%; NYHA III–V; eGFR ≥ 30 mL/min/1.73 m^2^	WHF or CV death	1.5	eGFR < 30 mL/min/1.73 m²	<60 (*n* = 1926) 41% ≥60 (*n* = 2816) 59, 35%	The effect of dapagliflozin on the primary and secondary outcomes did not differ by eGFR category or examining eGFR as a continuous variable.
EMPEROR reduced; 2020; Packer et al.;	3730	Empaglifozin vs. Pl.	LVEF ≤ 40%; NYHA IIIV; eGFR ≥ 20 mL/min/1.73 m^2^	WHF or CV death	1.3	eGFR < 20 mL/min/1.73 m²	<60 (*n* = 1978) 53, 2% ≥60 (*n* = 1746) 46.8%	Empagliflozin reduced the primary outcome and total HF hospitalizations in patients with and without CKD.
SOLOIST-WHF; 2021; Bhatt et al.	1222	Sotaglifozin vs. Pl.	18–85 years old; symptoms or sign of HF; type II diabetes; recent hospitalization for WHF.	Total WHF and CV death	0.75	eGFR < 30 mL/min/1.73 m²	<60 (*n* = 854) 69.9% ≥60 (*n* = 368) 30.1%	Sotaglifozin therapy resulted in lower total number of deaths from CV causes and hospitalizations or urgent visits for HF than placebo even in patients with CKD across the full range of proteinuria.
**Angiotensin Receptor Neprylisin Inhibitors**								
PARADIGM-HF; 2014; Solomon et al.	8442	Enalapril vs. Sac/Val	LVEF ≤ 40%; NYHA III–V; eGFR ≥ 30 mL/min/1.73 m^2^	CV death or HFH	2.25	eGFR ≤ 30 mL/min/1.73 m²	<60 (*n* = 3061) 36.2% ≥60 (*n* = 5338) 63.2%	Compared with enalapril, sacubitril and valsartan led to a slower rate of decrease in the eGFR and improved CV outcomes, even in patients with CKD.
PARAGON-HF; 2019; Solomon et al.	4822	Sac/Val vs. Valsartan	LVEF ≥ 45%; NYHA III–V; eGFR ≥ 30 mL/min/1.73 m^2^	CV death or HFH	2.92	eGFR ≤ 30 mL/min/1.73 m²	<60 (*n* = 2341) 48.5% ≥60 (*n* = 2454) 50.9%	Sacubitril–valsartan did not result in a significantly lower rate of total HFH and death from CV causes both in patients with CKD and without CKD.
**Agents Considered in Selected HFrEF Patients**								
SHIFT; 2012; Bohrer et al.	6558	Ivabradine vs. Pl.	LVEF < 35%; synus rhythm; Heart rate > 70 bpm	CV Death or HFH	1.9	Sever renal disease	<60 (*n* = 1579) 24.07% ≥60 (*n* = 4581) 69.85%	Ivabradine significantly reduced the combined primary end point of CV mortality or HFH compared with pl. The incidence of the primary end point was similar in both patients with (CKD stages 3–5) and without CKD.
VICTORIA; 2020; Armstrong et al.	5050	Vericiguat vs. Pl.	LVEF < 45%; NYHA III–V; recent hospitalization; eGFR 15 ≥ mL/min/1.73 m^2^ (no more than 15% of subjects with an eGFR in the 15 L/min/1.73 m2 to 30 mL/min/1.73 m^2^ range).	CV Death or HFH	0.8	eGFR < 15 mL/min/1.73 m²	≤30 (*n* = 506) 10% >30 ≤60 (*n* = 2118) 41.94% >60 (*n* = 2335) 46.23%	Vericiguat reduced the primary composite endpoint of CV death or HFH across all eGFR spectrum. the beneficial effects of vericiguat were similar in patients with and without WRF.
GALACTIC-HF; 2021; Teerlink et al.	8256	Omecamtiv /Mecarbil vs. Pl.	LVEF ≤ 35%; symptomatic chronic HF	CV Death or HFH/WHF	1.8	eGFR < 15 mL/min/1.73 m²	NA	Lower incidence of HF event or death from CV causes in the omecamtiv mecarbil arm compared with placebo.

ACM: all-cause mortality; CKD: chronic kidney disease; CV: cardiovascular; eGFR: estimated glomerular filtration rate; HF: heart failure; HFH: hospitalization for heart failure; Pts: patients; LVEF: left ventricular ejection fraction; NYHA: New York Heart Association; pts: patients; NA: not available; Pl.: placebo; and WRF: worsening renal function.
